# Re-Configuring Identity Postpartum and Sustained Abstinence or Relapse to Tobacco Smoking

**DOI:** 10.3390/ijerph16173139

**Published:** 2019-08-28

**Authors:** Tracey J. Brown, Linda Bauld, Wendy Hardeman, Richard Holland, Felix Naughton, Sophie Orton, Michael Ussher, Caitlin Notley

**Affiliations:** 1 Norwich Medical School, University of East Anglia, Norwich NR4 7TJ, UK; 2 Usher Institute, College of Medicine and Veterinary Medicine, University of Edinburgh, Edinburgh EH8 9AG, UK; 3 School of Health Sciences, University of East Anglia, Norwich NR4 7TJ, UK; 4 Leicester Medical School, University of Leicester, Leicester LE1 7RH, UK; 5 Division of Primary Care, University of Nottingham, Nottingham NG7 2RD, UK; 6 Population Health Research Institute, St George’s, University of London, London SW17 0RE, UK; 7 Institute for Social Marketing and Health, University of Stirling, Stirling FK9 4LA, UK

**Keywords:** tobacco smoking, return to smoking, smoking prevention, pregnancy, postpartum, social identity, qualitative research

## Abstract

Relapse to smoking postpartum is a common and important public health problem. Difficulty in adjusting to a non-smoking identity is a key factor prompting relapse. However, postpartum relapse prevention interventions rarely focus upon offering support for identity change. We conducted an exploratory inductive analysis of a dataset from the Prevention of Return to Smoking Postpartum (PReS) study to understand identity constructs and experiences of pre- and postpartum women (smokers and ex-smokers), partners and health professionals. Data were obtained from 77 unique participants via focus groups, interviews, email or online questionnaires, and were analyzed by two researchers independently, using NVivo 12. Four main themes emerged reflecting identity transition from the pre- to the postpartum period: (i) Pregnancy and the categorization of smoking status; (ii) the disruption of motherhood and loss of self; (iii) adapting to a maternal non-smoking identity; and (iv) factors influencing sustained abstinence versus relapse to smoking. Postpartum relapse prevention interventions need to consider support for women, and the whole family unit, in adjusting to a new identity as a non-smoking mother. Smoking status should be revisited throughout pregnancy and into the postpartum period to aid the long-term integration of smoke-free behavior.

## 1. Introduction

Relapse to tobacco smoking among women who quit smoking for or during pregnancy is common. Up to 76% of spontaneous quitters return to smoking postpartum [1]. Relapse to smoking is a critical public health problem, as there are significant personal and wider societal health costs of a return to smoking [2,3,4]. Babies exposed to second-hand smoke are more likely to suffer from poor infant health outcomes [2]. There are also important psychosocial implications, as children of families where smoking behavior is the norm are more likely to become smokers themselves in later life, perpetuating a cycle of health inequality [5].

Identity, as a smoker or non-smoker, mothering identity, social identity and relationships are strongly connected to postpartum smoking relapse [6,7,8,9,10]. Despite this, neither smoking cessation nor relapse prevention interventions with pregnant/postpartum women have focused on identity or identity change. We define identity as in [11]: ‘Identity is a relatively stable concept, a cohesive narrative, yet simultaneously fluid and shifting, continually subject to influence at multiple levels, and idiosyncratic within certain circumstances. Identity, at least partly, is discursively produced, continually reformulated by the negotiated understanding of selfhood shared through interaction’. Aspects of identity are recognized constructs within health psychology [12], and behavior change techniques (BCTs) in relation to identity can be coded as intervention components [13].

There is a strong policy drive to reduce smoking cessation in pregnancy [14], and identity aspects of stopping and staying stopped from smoking may be important to emphasize within any support provided. We aimed to conduct an exploratory inductive analysis of a qualitative dataset taken from a wider study (the PReS study: Prevention of Return to Smoking Postpartum) [15], to understand identity constructs, and the experiences of adjusting simultaneously to a new maternal and non-smoking identity, in order to explore how this might influence the likelihood of maintaining smoking abstinence postpartum.

## 2. Materials and Methods

We report a qualitative analysis of data from the Prevention of Return to Smoking Postpartum (PReS) study, an early phase study to develop an intervention to maintain smoking abstinence postpartum. We sought the views of pregnant and post-partum women (smokers and ex-smokers), partners and health professionals. We used semi-structured interviews to explore participants’ perceptions of smoking relapse, barriers and facilitators for behavior maintenance, and views on any potential components of a smoking relapse prevention intervention [15]. In this study we applied a constructivist grounded-theory approach (where results are generated inductively, but with a consideration of the prior theory of existing knowledge on the topic to inform analysis) [16] to explore perceived identity and smoking behavior in the social context of the pre- and postpartum periods. 

This study was approved by West Midlands-Edgbaston National Health Service (NHS) Research Ethics Committee on 21st July 2017 (ref: 17/WM/0285). Participants gave written consent prior to the interview. Recruitment and data collection took place between September 2017 and September 2018 in the East of England (Norfolk, UK). Participants were recruited via health visitors, midwives, NHS Smokefree Norfolk, and children’s centers using posters, flyers and electronic advertisements. In addition to referral from services, researchers identified participants in person, from antenatal clinic waiting areas and children’s centers. Participants were also recruited through general media, community and social centers, and word of mouth.

Eligibility criteria for all participants included being aged 18 years or over and able to communicate in English, due to the in-depth nature of the interviews. Specific inclusion criteria for women were: Having quit smoking during, or in the 12 months before pregnancy; currently pregnant or having given birth in the last five years; and self-reported ex-smokers or smokers (relapsed to smoking). The inclusion criterion for partners was to be a partner to a woman meeting the eligibility criteria. Eligibility for health professionals were: To be working as part of the health visitor services; midwifery services; NHS Smokefree Norfolk or children’s centers. To reach a diverse sample, interviews were conducted as focus groups or one-to-one interviews, via telephone or in person, at a time and location convenient to the participants. Participants were also invited to comment electronically (via email, or anonymously via an online questionnaire) if unable or unwilling to attend an interview. We aimed to recruit approximately 74 mixed stakeholders purposively selected by subgroup (42 pregnant/postpartum women, 16 partners, 16 health professionals) and continuing to recruit until we believed we had achieved a sufficient range of views. We aimed to over-sample women and ex-smokers in particular, in order to test a prototype intervention with women for the PReS study [15]. 

Semi-structured interview schedules were developed with patient and public advisors (PPI). Interviews lasted 90 minutes, and participants were compensated with a £20 shopping voucher. Interviews were conducted by Tracey J. Brown and Caitlin Notley (trained qualitative researchers), and digitally audio-recorded. Data were transcribed verbatim, and analysis was facilitated using NVivo version 12 (QSR International Ltd, Warrington, UK). Tracey J. Brown and Caitlin Notley independently coded three transcripts, met to review codes, and agreed a general coding framework, which was then applied across all transcripts by Tracey J. Brown. All coding relating to identity was extracted for subsequent analysis. This further analysis used constructivist grounded theory to code all data extracts relevant to identity, informed by a theoretical consideration of identity as a narrative in life course development [11]. All data relating to identity was independently coded by Tracey J. Brown and Caitlin Notley and verified by discussion. Findings were validated with all authors and patient public involvement (PPI)representatives. 

## 3. Results

Data were obtained from 77 unique participants who took part in the PReS study [15] (Table 1). There were no distinguishable differences as a result of the different interview methods, however electronic data was less descriptive. Four main inductive themes were generated based on identity as a central theme; which reflected a narrative of identity change from pre- to postpartum. We focus primarily upon the experiences of women, supplemented with insights from health professionals and women’s partners. 

### 3.1. Pregnancy and Categorisation of Smoking Status

A strong theme unique to this population was anxiety around classification as a current ‘smoker’ or ‘non-smoker’ at the first maternity booking appointment. Women’s response to the question of their smoking status by a midwife was perceived as a crucial moment of assigned identity, which would influence them through their current pregnancy, to motherhood. Primary reasons given for desiring a pregnant ‘non-smoker’ status were concerns of judgement and stigma, and of pressure by health professionals or others, to change their behavior. Women expressed a desire to move from a stigmatized smoking ‘out-group’ to an accepted non-smoking ‘in-group’. Many participants held powerful, often moral, beliefs against smoking whilst pregnant:

*‘I certainly don’t want to judge anyone but I personally found there’s something that really makes you think okay, there’s a baby inside me. You know what I am putting into my body directly affects that’ (025 postpartum ex-smoker)*

There was mention of pride, conviction and resolve in remaining smoke-free. Some women indicated they felt impervious to relapse and were unwilling to admit any vulnerability to their new non-smoking identity:

*‘for someone like me right now, I feel very proud that I’m in the non-smoker box. I don’t want to be put back in the smoker box because I want to kind of resist being there’ (001 pregnant ex-smoker)*

Health professionals recognized that relapsing to smoking during pregnancy might be perceived as ‘taboo’, ‘a stigma’, and might be ‘embarrassing’ for women to admit. Maintaining a good patient relationship was perceived as paramount, and professionals realized patients’ concerns of judgment. However, they acknowledged this discord likely acted as a barrier to establishing the smoking status of women and their partners, and providing support:

*‘Like you say, people feel so judged and so dreadful but actually there’s hundreds of other mums and dads and partners that feel exactly the same way’ (009 smoke-free advisor)*

For most women the key motivator for quitting smoking was ‘for the baby’ and not to change their own smoking identity or health status. Women spoke of struggles in remaining smoke-free and meeting expectations of a non-smoker identity. Difficulties in adjusting to a pregnancy identity were evident, particularly in the cases of first pregnancies, unplanned pregnancy, or in the first trimester where the pregnancy was (in some opinions) less ‘real’. For others the final trimester was a particular challenge, with some anticipating relapse after birth, or giving themselves permission to return to smoking:

*‘So I stopped but for example, in my sleep, like I dreamt every single night that I’m taking—I’m smoking, so for me it was, waiting to give birth and have my first cigarette’ (027 postpartum relapser)*

This participant reflected on what smoking meant to her and her thoughts about ‘losing that part’ of her, which led to her relapse. For women with smoking partners, abstinence could be particularly challenging, in light of their partner’s views of their smoking identity:

*‘I’m doing the damage to myself but I’m not doing the damage to the baby’ (051 male partner)*

This sense of ‘distancing’ in some cases reflected partners’ perceptions of playing a relatively insignificant role during the pregnancy. Both women and partners spoke of barriers for partners in attending antenatal appointments, often due to employment. Some partners felt alienated, ‘a spare part’, even when attending appointments. Most participants felt the key focus was on the baby, and whilst this was appreciated, the parents could feel disconnected as a unit. Women could feel positioned as the go-between, relaying information from health professionals to their partner:

*‘I kept trying to say to him…smoking around me is still going to have an impact but because it was me saying it, not a professional or somebody who knows what they’re on about, he wasn’t really interested’ (023 postpartum ex-smoker)*

Pregnant women generally had few relapse prevention strategies, relying upon willpower and physiological changes reducing their smoking cravings. Hormones, mood swings and the ‘crazy journey’ of pregnancy could detrimentally affect coping. For women relapsing during pregnancy, feelings of guilt and remorse as a mother-to-be were described:

*‘I done it! So I took her choice away and that to me reads as wrong and it’s not supposed to be that you take the choice away from your own kid. She’s trying to grow and be healthy inside there and she’s got no other choice what she breathes’ (028 postpartum relapser-initial relapse in pregnancy)*

### 3.2. Disruption of Motherhood and Loss of Self

Shortly after birth, women felt particularly vulnerable to relapse, or indeed did relapse. Vulnerability was due to perceived factors including a reduction in intensive health service support, leaving ‘the midwife bubble’ and entering the ‘scary zone’, and partners or family members smoking. Of greatest significance, risk of relapse increased due to feelings of dissociation and detachment from the baby on leaving a pregnancy identity:

*‘I was like oh, I’ve given birth now, ooh, I could smoke if I want to’ (039 postpartum relapser)*

Feelings of loss and disruption were strong and recurring themes. Whilst many women expressed the joy of having a baby, they simultaneously felt a loss of themselves and their pre-birth identity:

*‘it’s such a massive lifestyle change and what I felt, although I’m absolutely over the moon and I’m so happy being a mummy, you get really lonely…you feel like you’re struggling and it’s never ending’ (004 postpartum ex-smoker)*

The abrupt change to parenthood was perceived by many to be disruptive, an acute interruption to their former lives. Women spoke intensely of feelings of loneliness and low mood, particularly on the return of partners to work and a reduction of family visits after the initial excitement of the new baby. Participants were affected by feelings of stress at having a baby to care for, and in some cases, perceptions of a new mundane regime. Smoking was frequently perceived as a coping response for these stressors. Women could be sentimental when reflecting on their pre-pregnancy smoker identity:

*‘when I was younger, like even couple of years ago, I’d just go down the pub, you know, have a drink, have a smoke in the sun, it was lovely. It was lovely’ (044 postpartum ex-smoker)*

Some women expressed resentment at losing their former identity, particularly in light of partners or family members:

*‘we’ve got to be the ones about health and serenity and all this—where the dads can just carry on normal life’ (034 postpartum ex-smoker)*

Women felt conflicted and under pressure to be ‘perfect’. They perceived an expectation to stay smoke-free, having achieved this during pregnancy. However, women could struggle to conceptualize themselves as non-smokers, ‘once you’re a smoker, you’re always a smoker’. Smoking for many women and also partners, was an integral part of their identity prior to parenting, thus relapse could be positioned as part of regaining the previous ‘lost’ identity:

*‘It was very easy to go backwards to what I knew…I have such a limited social life and you need something just to take away from the nappies and the bottles and everything. You do it every day so you need something else’ (028 postpartum relapser)*

*‘her mum…said she’ll have her the night. It’s often we’ve turned around and said well what did we do before we had a kid? And I’ve gone and said bloody this! And we’re sitting outside and having a fag or sitting in the kitchen having a fag’ (029 male partner)*

### 3.3. Adapting to a Maternal Non-Smoking Identity

Further into the postpartum period, it became easier for women to adapt to a maternal non-smoking identity, wanting to do the ‘right thing’ for their child. There was an acknowledgement of responsibility, particularly for the baby, and in some cases for the wider family. Some women spoke of their longevity, hopes for seeing their child into adulthood, and perhaps have children of their own. These feelings of accountability and the extension of their perceived identity to include their baby, drove both adjustment to a maternal identity, and a focus upon maintaining positive health behavior change:

*‘It makes you feel like a mum as well because you’re protecting your baby by something that you can do… there’s other things that you couldn’t prevent, but if this is something that I can prevent, I’m going to do it’ (064 postpartum ex-smoker)*

This strong maternal nurturing instinct, and status as a ‘mum’, acted as a key motivator to remain smoke free. Concern of the link between smoking and an increased risk of sudden infant death syndrome (SIDS—also known as cot or crib death) was central. Some women also referenced the increased likelihood of their child becoming a smoker if they witnessed their smoking. 

These concerns, and the passing of time further into the postpartum period, aided a shift in smoking attitude, and in turn, assisted the transition of identity from smoker to non-smoker:

*‘I hate it now, I absolutely hate it now. I’m shocked that I used to be a smoker’ (064 postpartum ex-smoker)*

Most women used willpower and an imagining of future outcomes for their baby, to help them stay smoke-free postpartum. Many were also driven by a desire to be in control of their new lives and live without ‘guilt’. Women who were successful in remaining smoke-free appeared to have developed more strategies to maintain smoking abstinence, and more coping skills for general parenthood. Knowledge of coping skills was largely gained through self-help techniques. These techniques were varied, ranging from practical approaches, such as a jar to save money from not smoking, to psychological techniques, including boosting mood, reducing stress, and reflecting on their reasons for their previous smoking. Some women capitalized on their change in lifestyle to break these smoking habits. It was important to acknowledge that days could be ‘tough’, and there was a deliberate avoidance of putting themselves under the pressure of conforming to societal expectations. There were some references to the importance of maintaining a sense of self:

*‘to the woman who hasn’t been herself for a while—stay hopeful, that spark will reignite soon. This weight will have itself lifted off your tired shoulders soon…you’ll be back’ (059 postpartum ex-smoker)*

This mother was referring to an online poem which struck a chord with her. Maternal status and focus on the baby, in some instances provided pivotal moments for supporting smoking abstinence:

*‘it was something that I could kind of cling onto, almost like a bit of a life raft at times where it was remembering that I can deal with stressful situations without having to go and have a cigarette. And I remember thinking at the time; stress isn’t something that just suddenly happened to me at 16 when I had my first cigarette…at his age, there will be situations that are a bit stressful, he doesn’t need to have a cigarette to cope with them, does he? So why do I?’ (058 postpartum ex-smoker)*

This participant makes reference to clinging on to her strategy like a ‘life raft’, acknowledging how challenging it was for her to stay abstinent.

### 3.4. Factors Influencing Sustained Abstinence versus Relapse to Smoking

A range of factors influenced the likelihood of integrating smoke-free behavior and maintaining long-term abstinence. Partner influences were frequently raised. Staying smoke-free was easier if living with a supportive partner, particularly where partners were non-smokers or ex-smokers. In addition to encouraging women’s continued smoking abstinence, providing general support for women’s welfare and support as a father in caring for the baby were perceived as significant:

*‘(my partner) will put him to bed so it just gives me an extra like twenty minutes to chill out…it helps loads doesn’t it’ (062 postpartum ex-smoker)*

Enabling women to have more time to themselves helped them to retain their pre-pregnancy identity and to integrate the new identity of a maternal non-smoker. Similarly, the role of family, friends and wider society were influential, both directly, and more indirectly, by promoting a shift in perceived smoking norms.

*‘I know I can’t smoke in my car and I wouldn’t, I don’t think I would smoke now at work because it’s now a non-smoking site’ (065 postpartum ex-smoker)*

Whilst some women were able to integrate their smoke-free behavior, for others, adapting to a non-smoking identity was unmanageable. This was particularly the case where women relied heavily upon willpower as a relapse strategy. Particular risky times for relapse postpartum were social situations, stopping breastfeeding, or returning to work. These times presented openings for women to revisit their pre-pregnancy smoking identity. Having smoking family members, friends, or more crucially a smoking partner, had detrimental effects:

*‘the whole time through all my pregnancies, he never gave up and we were walking somewhere and he had a cigarette and I said oh just let me try a bit…as soon as I had a toke, then that was it, I was smoking again’ (056 postpartum ex-smoker)*

This ex-smoker was describing a previous postpartum relapse. There was a sense of being readily hooked back into nicotine dependence. Our sample generally perceived tobacco smoking as an ‘addiction’ that was difficult to control and had formed part of their previous identity. For some, this contributed to a disconnection from their identity as a maternal non-smoker, increasing the likelihood of a smoking relapse:

*‘I’m like oh back in the day I used to be such a little lush and smoke fags. So in my head I’m like well this is what I do’ (059 postpartum ex-smoker)*

With hindsight, many women reflected that it might have been beneficial if their smoking status at the first midwife booking appointment was re-visited. For many it seemed to be difficult for them to raise this subject themselves, and they looked to health professionals to prompt this conversation in a non-judgmental way:

*‘there was never a conversation about smoking in any of my appointments apart from that very first one. So if they knew that I was a smoker in the past, it should be highlighted and then they could maybe go—this has been quite tough, you know, have you felt the urge to start smoking again? Have you started smoking again? Is there anything we can do to help you stay smoke-free and keeping the smoking in the forefront of the conversation because at the moment it’s a one tick box and never mentioned again’ (034 postpartum ex-smoker)*

Identity is co-constructed in relationship with others, health professionals, family and partners. From the defining moment at the first midwife booking appointment, all stakeholder groups perceived opportunities to further engage a woman’s partner or wider support:

*‘if you visit a family and there’s a dad there and you ask them a question about how they are, they almost like nearly want to fall off their chair because someone’s actually given them some speaking time’ (011 health visitor)*

*‘So encouraging them to quit and kind of, yeah letting them know at that point, you know to get their partners thinking about e-cigarettes as well, would be a good time’ (022 male partner)*

## 4. Discussion

Our study explored the identity constructs and experiences of adjusting to a new maternal, non-smoking identity. Four inductive themes were generated based on identity as a central theme. We theorized identity to be fluid and shifting, and our findings clearly demonstrate that changes in the perception of identity as a mother and a non-smoker, reflected the transition from the pre- to the postpartum periods. 

For both new mothers, and those who had previously had children, pregnancy acted as a powerful motivator to quit smoking. By quitting smoking in pregnancy, women experienced a positive identity change and the sense of movement from a stigmatized ‘out-group’ to an accepted ‘in-group’. Women were eager to classify themselves as a ‘non-smoker’ at their first midwife booking appointment. For some women, this classification generated a potentially misplaced feeling of being impervious to relapse after the birth of the baby, whereas other women were anticipating a return to smoking. Uncoupling from the baby at birth, and the disruption of entering motherhood, increased this vulnerability to relapse. Our analysis revealed loss and disruption as strong themes of identity change on entering the postpartum period. Smoking for many was a fundamental part of their pre-parental identity, and consequently relapse could be viewed as part of regaining this ‘lost’ identity.

Whilst some women failed to adapt to motherhood and relapsed, others adapted, re-configuring identity and remaining abstinent. Integration of smoke-free behavior was heavily influenced by coping strategies, the support of partners, and wider social support. Many women wanted health professionals to discuss their smoking status in later pregnancy and postpartum, and to offer assistance to them and/or their partners to maintain long-term smoking abstinence. 

A potential weakness of this study was the low proportion of partners who we were able to recruit to participate. We also included a high proportion of ex-smokers relative to smokers. We aimed to oversample ex-smoking women to test a prototype intervention to prevent relapse as part of our wider PReS study [15], and have included the views of both smokers and ex-smokers in this current analysis. All participants were recruited within one UK county (Norfolk). However, a wide recruitment strategy and broad eligibility criteria enabled a variety of participants to be included, and we believe these findings would be relevant to other areas and contexts. Gathering qualitative data from a wider sample of women and partners in other countries would be of interest to explore the transferability of these findings. We offered participants the option to participate in the study as a group, or individually in-person or via telephone or electronically, to maximize participation. However, we did not formally consider the influence of these different types of methods on participant responses. 

Strengths of this study include independent coding of data by two experienced qualitative researchers and the validation of findings with all authors and PPI groups. We included a wide variety of stakeholders to triangulate data sources: Pregnant and postpartum women, partners and various health professionals. We offered interviews both in-person and over the telephone, and offered reply electronically and/or anonymously to broaden reach. 

Our results reinforce and advance findings from the literature, showing that difficulties in adjustment to identity as a mother and a non-smoker are key influences in return to smoking postpartum [6,7,8,9,10]. Others have found that pregnancy and the desire for optimal health of the fetus act as key extrinsic motivators to quit smoking, and an intention to quit smoking only during pregnancy to prompt relapse [6,7,10]. Smoking in pregnancy is acknowledged as generating feelings of guilt, fears of judgment, and disapproval from society [10]. We found both the motivation of pregnancy and the perception of stigma to be key influences in women’s eagerness to classify themselves as ‘non-smokers’. Self-reported smoking status and national statistics, such as smoking status at the time of delivery, are susceptible to underreporting, with more women in deprived areas going undetected [17,18,19]. This dichotomous categorization as ‘non-smoker’ versus ‘smoker’ limits collection of data from recent smoking quitters, and minimizes opportunities to support sustained abstinence and prepare for the postpartum period. 

Women were perhaps particularly susceptible to relapse after the birth of their baby due to a longing to regain their former pre-pregnancy identity, low confidence in remaining quit, and stresses of caring for a new infant [6,7,9,10,20]. There was a misaligned belief that smoking provided relief from the stresses of parenthood, which has been identified as a key factor in postpartum relapse [7]. Participants who were successful in remaining abstinent appeared to have developed more relapse prevention strategies, a phenomenon which extends to general smoking quitters [21]. A desire to shift to the identity of a non-smoker was driven by a motivation to be in control, to lead an overall healthier family lifestyle, and to act as a parent role model, a ‘better mum’ [7,10]. The capacity to successfully reframe identity and maintain abstinence was facilitated by the ability to retain a sense of former identity prior to motherhood, focusing on non-smoking aspects.

Identification of self as a role model and valued self-identity are recognized behavior change techniques, which might be used to support smoking cessation during and after pregnancy in future interventions and clinical practice [13]. Currently these techniques are underutilized in postpartum relapse prevention interventions [22]. The ability to maintain a non-smoking identity and smoking abstinence in the long-term is affected by interaction with significant others, in particular partners, who are often less involved in ante- and postnatal appointments [9,10,11,23,24]. Interventions are generally focused on the individual, whereas we found a need for intervention at the interpersonal level, to include close friends and family (notably partners), and wider society. 

Health professionals (health visitors, midwives) as part of society, might aid the construction of a new non-smoking identity. However, professionals are often concerned about raising/repeatedly raising the subject of smoking, due to the potential negative impact on the therapeutic relationship [23]. Conversely, a novel and important finding of this study was that women had a strong desire for health professionals to revisit their smoking status in later pregnancy and postpartum, so as to provide validation, encouragement and assistance if needed. Each health appointment offers a ‘teachable moment’ to review women’s smoking status, their beliefs and concerns, and to offer support [25,26], in line with the ‘Making Every Contact Count’ policy initiative [27]. NICE guidance recommends carbon monoxide testing for smoking status at maternity booking and subsequent appointments [28,29], offering opportune moments to normalize the discussion of smoking during and after childbirth. Embedding discussion as part of routine appointments enables a patient-centered approach, in harmony with the construction of a non-smoking identity. Health professionals should be enabled with additional time, tools and skills for this to be successful, and more meaningful than purely a prescribed method to record smoking status [23,30]. Future research is needed to test the success of interventions incorporating identity change within the wider social support network and within the context of standard care. Investigating the influence of different sociodemographic characteristics and length of quit prior to pregnancy on the likelihood of relapse postpartum, would also be beneficial. 

## 5. Conclusions

Our analysis suggests that interventions to support postpartum relapse prevention must critically include support for women in adjusting to a new identity as a non-smoking mother as women transition from the pre- to the postpartum periods. Interventions that do not acknowledge the struggle of managing disruption to the narrative flow of identity may be less effective. Identity is co-constructed with others, and the role of social support, particularly from partners, must be considered as part of identity adjustment. Health practitioners should make opportunities to include the wider family unit, and to revisit smoking status at every opportunity. 

## Figures and Tables

**Table 1:** Participant sample.

Participants	Interviews Completed	Online/Email Feedback
Postpartum smokers (relapsed to smoking)	7	2
Postpartum ex-smokers	19	6
Pregnant smokers (relapsed to smoking)	5	0
Pregnant ex-smokers	9	4
Partners	7	2
Did not specify	0	4
Health professionals (midwives, health visitors, stop smoking advisors, children’s center staff)	12	0
Subtotal	59	18
Total		77
